# Molecular Basis of Neuronal Autophagy in Ageing: Insights from *Caenorhabditis elegans*

**DOI:** 10.3390/cells10030694

**Published:** 2021-03-21

**Authors:** Georgios Konstantinidis, Nektarios Tavernarakis

**Affiliations:** 1 Institute of Molecular Biology and Biotechnology, Foundation for Research and Technology-Hellas, 70013 Heraklion, Greece; georgios_konstantinidis@imbb.forth.gr; 2 Department of Basic Sciences, School of Medicine, University of Crete, 70013 Heraklion, Greece

**Keywords:** ageing, autophagy, *Caenorhabditis elegans*, neurodegeneration, macroautophagy, neuronal autophagy

## Abstract

Autophagy is an evolutionarily conserved degradation process maintaining cell homeostasis. Induction of autophagy is triggered as a response to a broad range of cellular stress conditions, such as nutrient deprivation, protein aggregation, organelle damage and pathogen invasion. Macroautophagy involves the sequestration of cytoplasmic contents in a double-membrane organelle referred to as the autophagosome with subsequent degradation of its contents upon delivery to lysosomes. Autophagy plays critical roles in development, maintenance and survival of distinct cell populations including neurons. Consequently, age-dependent decline in autophagy predisposes animals for age-related diseases including neurodegeneration and compromises healthspan and longevity. In this review, we summarize recent advances in our understanding of the role of neuronal autophagy in ageing, focusing on studies in the nematode *Caenorhabditis elegans*.

## 1. Introduction

### 1.1. Mechanisms of Autophagy

Autophagy is an evolutionarily conserved process enabling cells to regulate a plethora of catabolic needs. Under physiological conditions, autophagy serves as a quality control mechanism in order to maintain cell homeostasis. Importantly, in response to extracellular or intracellular stress, autophagy is employed to degrade and recycle macromolecules such as misfolded or aggregated proteins as well as to eliminate dysfunctional organelles or invading pathogens [1,2,3]. Macroautophagy, microautophagy and CMA (chaperone-mediated autophagy) are the three morphologically and mechanistically distinct types of autophagy. However, all three converge towards the delivery of cargo to the lysosome for degradation.

In microautophagy, cytoplasmic components enter the lysosome through a direct invagination of the lysosomal limiting membrane. Membrane fission results to the release of a microautophagic body into the organelle lumen for degradation [4]. CMA involves the selective recognition of a substrate based on the KFERQ-like motif by a cytosolic chaperone, the HSC70 (heat shock cognate 71 kDa protein) and cochaperones [5]. Subsequently, unfolding of the substrate and translocation via the receptor LAMP2A (lysosome-associated membrane protein type 2A) leads to the degradation of the substrate.

**Figure 1:**
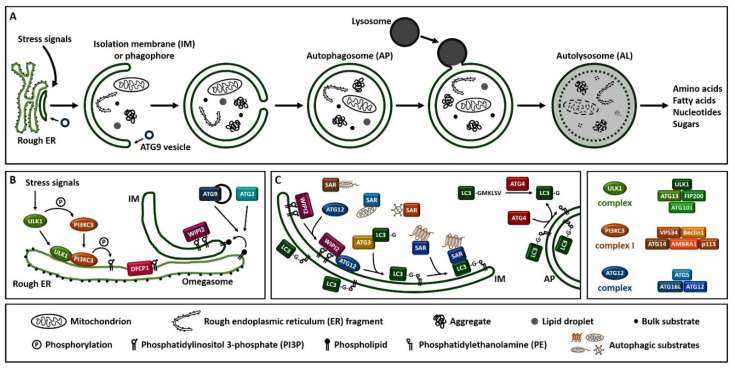
Overview of the autophagy process in human. (**A**). Schematic representation of autophagosome formation and cargo degradation. Complete autophagosomes may fuse with endosomes to form amphisomes, which further fuse with lysosomes. (**B**). Omegasome and isolation membrane generation. (**C**). Isolation membrane expansion, LC3 processing and autophagic substrate sequestration. Additional membrane sources may contribute to isolation membrane formation and expansion. SAR: selective autophagy receptor, LC3-GKLSV: pro-LC3, LC3-G: LC3-I, LC3-G-PE: LC3-II.

Macroautophagy (hereafter referred to as autophagy), is a process that enables cells to recycle and degrade intracellular contents. Hallmark of autophagy is the de novo formation of a double-membrane vesicle, termed the autophagosome, which sequesters and engulfs cytoplasmic material (Figure 1A). Subsequently, autophagosome matures and fuses with a lysosome to form an autolysosome, leading to the exposure of the autophagic cargo to the lysosomal hydrolases for digestion [6,7,8]. Autophagosome formation is driven by the orchestrated action of approximately 20 ATG (autophagy-related) proteins, whose role is essential for autophagic delivery of cargo to the lysosome or vacuole in yeast (Figure 1B,C) [9,10,11,12,13]. Nutrient and energy sensing pathway hubs, including mTOR (mechanistic target of rapamycin) and AMPK (AMP-activated protein kinase), converge to the ULK1 (Unc-51-like kinase 1) complex [ULK1, ATG13, FIP200/RB1CC1 (200 kDa FAK family kinase-interacting protein/RB1-inducible coiled-coil protein 1) and ATG101] for autophagy initiation (Figure 1B). ULK1 complex triggers the nucleation of an autophagosome membrane precursor, termed isolation membrane or phagophore by phosphorylating members of the PI3KC3 (class III phosphatidylinositol-3 kinase) complex I [VPS34 (vacuolar protein sorting 34), Beclin 1, ATG14, AMBRA1 (activating molecule in Beclin 1-regulated autophagy protein 1) and general vesicular transport factor (p115)]. PI3KC3 complex I induces the local production of PI3P (phosphatidylinositol-3-phosphate) at a characteristic endoplasmic reticulum structure called the omegasome. Two PI3P effector proteins the WIPI2 (WD repeat domain, phosphoinositide interacting 2) and the DFCP1 (zinc-finger double FYVE-containing protein 1) are recruited to the omegasome via interaction with their PI3P-binding domains. Locally accumulated WIPI2 directly binds and recruits ATG12 (ATG12-ATG5-ATG16L1) complex and catalyzes the conjugation of the C-terminal glycine of ATG8 family proteins, [including LC3 (microtubule-associated protein light chain 3) and GABARAP (γ-aminobutyric acid receptor-associated protein)] to the phagophore-resident lipid PE (phosphatidylethanolamine) (Figure 1C). The lipidated, thus membrane-bound LC3 (called LC3-II), characteristic signature of autophagic membranes, is required for elongation and closure of the phagophore. ATG4, the sole ATG protease, processes nascent ATG8 to expose the C-terminal glycine residue [14]. ATG8 protein family members interact with autophagy receptor proteins or SARs (selective autophagy receptors) bearing LIRs (LC3-interacting regions) in order to sequester the cytoplasmic material destined for lysosomal degradation [15]. The endoplasmic reticulum (ER) and the majority of cellular membranes have been proposed to contribute membrane material for the elongation of the autophagosomal membrane [16,17]. ATG2 mediates phospholipid transfer from ER or other donor membrane sources to an ATG9-positive vesicle/phagophore. ATG9, the sole transmembrane ATG protein possesses membrane-bending properties. It facilitates autophagosomal membrane expansion acting as a scramblase. Specifically, ATG9 transports phospholipids from the cytoplasmic to the luminal leaflet of the isolation membrane [18,19,20,21,22]. ESCRT (endosomal sorting complexes required for transport) machinery facilitates phagophore closure, a process involving membrane fission of the inner and outer membrane at the phagophore edge [23,24]. After phagophore closure, the complete autophagosome undergoes maturation by dissociation or removal of most ATG proteins, including ATG8 by ATG4 protease, as well as PI3P by MTMR (myotubularin-related) phospholipid phosphatase from the autophagosome surface [25]. ATG8 family members, tethering factors, Rab (Ras-associated binding) GTPases, and SNARE (soluble *N*-ethylmaleimide-sensitive factor-attachment protein receptor) proteins act in concert to mediate fusion of autophagosomes with lysosomes [26]. Finally, the lysosomal acidic hydrolases digest the sequestered autophagic cargo and salvaged building blocks including amino acids, fatty acids, nucleotides and sugars are recycled back to the cytoplasm in order to be used again by the cell.

Autophagy is considered to have primarily cytoprotective and pro-survival functions. Therefore, as a critical cellular process it needs to be tightly regulated in order to respond and adapt appropriately to various cellular stress signals and insults. Several mutations in *ATG* genes have been identified and associated with human genetic disorders, developmental abnormalities, immune diseases, cancer and neurodegeneration, demonstrating a role for autophagy in animal health [27,28].

### 1.2. Neuronal Autophagy

As anticipated, autophagy is indispensable for neuronal survival and maintenance of neuron homeostasis. Genetic studies have demonstrated that conditional ablation of the core autophagy genes *atg5* and *atg7* in the nervous system of mice leads to accumulation of ubiquitin-containing inclusion bodies disrupting neuronal function and causing progressive neurodegeneration [29,30]. Autophagy in hypothalamic neurons is required specifically for the regulation of nutrient uptake, energy balance, obesity as well as ageing [31,32,33,34]. Hippocampal neurons of fragile X syndrome mice model exhibit reduced levels of autophagy. Activation of autophagy partially rescues the aberrant dendritic spine morphology, mGluR-LTD (metabotropic glutamate receptor-dependent long-term depression) and cognition in those mice [35]. Knockout studies have demonstrated an essential role for autophagy in embryonic neurogenesis and mutations in autophagy genes lead to developmental delay, cognitive decline, and functional deficits in childhood [36,37,38,39,40,41,42,43,44,45,46,47,48,49]. In addition, autophagy plays a role in the differentiation of adult NSCs (neuronal stem cells) [50,51,52,53,54,55,56,57]. Furthermore, specific processes involved in neuronal plasticity such as axonal growth, synaptic assembly, and dendritic spine formation and pruning are linked to autophagic activity and accompanied by cognitive deficits, anxiety-like behaviors, autism-like behaviors and memory deficits [35,58,59,60,61,62,63,64,65]. Given the contribution of autophagy in neurogenesis and neuronal plasticity, it is apparent that pharmacological or genetic induction of autophagy might be a means of treatment and therapy for disorders like depression, bipolar disorder, and schizophrenia [66,67,68,69,70,71,72,73,74,75,76,77]. In contrast, the lysosomal acidification inhibitor bafilomycin A1, which blocks autophagosome-lysosome fusion and therefore autophagic flux, has antidepressant effects in rats exposed to chronic unpredictable mild stress [78]. In that case, autophagy inhibition seems to be advantageous.

Neurons possess unique structural and functional characteristics. Cellular processes have to be tightly and differentially regulated in a spatiotemporal manner. For example, neuronal soma is often located far away from the synapses. Therefore, specialized processes restricted to the microenvironment of the synapse ensure proper synaptic transmission. Synaptic activity has been linked to autophagy in molecular and vesicular level [61,79,80,81,82,83,84,85]. Constitutive de novo autophagosome biogenesis occurs at the axon terminal and upon completion, autophagosomes fuse with late endosomes and/or lysosomes. In distal axons of primary DRG (dorsal root ganglion) neurons, autophagosomes seem to be generated at DFCP1-positive subdomains of the endoplasmic reticulum, distinct from ER exit sites [86]. Moreover, plasma- or mitochondrial-derived membranes were not incorporated into nascent autophagosomes. A minor population appears to arise from pre-existing autophagosome rings. The authors suggest that autophagosome rings may sometimes nucleate other smaller autophagic structures. Subsequently, autophagosomes are transported along the axonal microtubules toward the soma, in a dynein-dependent manner [87,88,89,90,91,92]. In transit, they fuse with additional lysosomes, arriving at the soma as fully competent degradative organelles. In general, autophagosome biogenesis events are enriched distally. However, autophagosomes form infrequently in dendrites, the soma, or midaxon [86].

Presynaptic autophagosomes engulf synaptic vesicles and therefore autophagy regulates neurotransmission by controlling the pool of the synaptic vesicles and neurotransmitter release [60,79]. Pharmacological activation of autophagy reduces the number of synaptic vesicles. Loss of autophagy increases evoked dopamine release in mice. BDNF (brain-derived neurotrophic factor) stimulates retrograde motility of autophagic compartments positive for the receptor of BDNF, TrkB (tropomyosin receptor kinase B) [93]. The phosphorylated endocytic adaptor endophillin A is enriched at the presynaptic terminals and promotes autophagy by generating highly curved membranes, where core autophagy proteins are recruited to form autophagosomes [82,83]. The lipid phosphatase synaptojanin 1, implicated in synaptic vesicle trafficking, is also required for autophagosome biogenesis at presynaptic terminals [61]. The scaffolding protein bassoon, localized to the presynaptic nerve terminals as well, sequesters ATG5 and inhibits presynaptic autophagy [60]. Mitophagy targets ubiquitinated mitochondria in synapses; thereby it may regulate local energy supply or calcium buffering capacity [94].

Predominant destination for autolysosome cargo degradation is the cell body, where lysosomes with high proteolytic activity reside [95,96]. Inhibition of lysosomal activity leads to accumulation of autophagosomes specifically within the soma, and not in the axon or dendrites [97]. Sequestration of mitochondria fragments have been captured into a subpopulation of autophagosomes [98]. Interestingly, an alternative mechanism for eliminating protein aggregates and mitochondria when autophagy is compromised is the secretion of large vesicles called exophers by several neuronal cell types, including dopaminergic and sensory neurons [99,100]. Extruded exophers are engulfed by neighboring cells for lysosomal degradation or released into the extracellular milieu. These finding suggest that neuronal cells do not necessarily degrade their own aggregates or damaged organelles, introducing a possible mechanism for the prion-like propagation phenomenon. From a pharmacological point of view, on one hand, induction of the release of toxic cargo outside of the cells will probably relieve the neurons from toxic loads but increase the propagation of prion-like pathologies. On the other hand, inhibition of the jettison of aggregates or damaged organelles, will probably prevent the pathology from spreading but will increase the toxic load into the neurons with possible detrimental consequences.

Postsynaptic autophagy plays a role in synaptic plasticity and synaptic strength, which are fundamental processes underling learning and memory [101,102,103,104]. Deletion of WDR45 (WD repeat domain 45) protein at the central nervous system leads to deficits in learning and memory in mice [105]. In LTP (long-term potentiation), low-dose activation of NMDARs (*N*-methyl-D-aspartate receptors) leads to an increase in AMPAR (α-amino-3-hydroxy-5-methyl-4-isoxazolepropionic acid receptor) internalization [106]. Conversely, in LTD (long-term depression), activation of NMDARs results in AMPARs degradation in hippocampal neurons. BDNF suppresses autophagy in cortical and hippocampal neurons, facilitating LTP and the persistence of memories in mice through stabilization of the postsynaptic scaffold proteins PICK1 (protein interacting with C kinase 1), PSD-95 (postsynaptic density protein 95) and SHANK3 (SH3 and multiple ankyrin repeat domains 3) [107]. Upon denervation of neuromuscular junctions, GABA_A_Rs (gamma-aminobutyric acid type A receptors) are selectively sorted via endocytosis from the postsynaptic membrane of muscle cells to autophagosomes, whereas acetylcholine receptors in the same cells do not localize in autophagosomes [108].

Interestingly, non-canonical roles of autophagic machinery and ATG proteins have been linked to neuronal biology. ATG16L localizes on hormone-containing dense-core vesicles through interaction with RAB33 and participates in hormone secretion from neuroendocrine PC12 cells independently of its role in autophagy through RAB33 [109,110]. LANDO (LC3-associated endocytosis) regulates amyloid-β clearance in microglia [111]. The inflammatory cytokine IL-1β (interleukin-1β) is secreted via an unconventional autophagy-mediated secretory pathway in human neutrophils [112].

In conclusion, accumulating evidence support the prominent role of autophagy in the regulation of a wide range of cellular functions in neurons which are further translated in behavioral outputs [85,113].

### 1.3. Autophagy in Ageing

Ageing, the time-dependent decline in cellular, tissue and organismal functions occurs in all metazoan organisms. Genetics play fundamental role in controlling ageing [114]. Numerous mutations that either extend lifespan or accelerate age-related decline have been characterized. Studies in those mutated genes revealed specific signaling pathways and cellular machineries conserved among species that regulate ageing. Accumulating evidence supports autophagy as a critical regulator of lifespan [115,116]. Caloric restriction and mTOR inhibition, two interventions known to extend lifespan, induce autophagy [116]. Autophagic activity and efficiency declines with age in diverse organisms. From *C. elegans* and rats to primary human cells, ageing reduces the capacity of lysosomal proteolysis compared to their younger counterparts [117,118]. Reduced expression of several *ATG* genes upon ageing is documented in several organisms including *Drosophila* and rodents [119,120,121]. In *Drosophila*, loss-of-function mutations of *Atg7* and *Atg8* genes reduce lifespan, increase sensitivity to stress and promote neuronal accumulation of ubiquitin-positive aggregates [119,122]. Mice exhibit an age-dependent decrease in autophagosome numbers [123]. In addition, genes important for autophagosome-lysosome fusion such as *LAMP2*, show reduced expression upon ageing [124]. In human brain, *ATG5*, *ATG7* and *BECN1* are down-regulated during normal ageing [125]. Individuals with age-associated neurodegeneration diseases possess autophagy gene polymorphisms and exhibit reduced autophagy [126,127,128,129]. Since impairment of autophagy predisposes organisms to age-related diseases, such as neurodegeneration, genetic or pharmacological restoration of autophagy may be utilized for improving ageing-related diseases [115].

Oxidative stress, DNA damage, telomere shortening and inflammation play prominent causative role in ageing [130,131,132,133]. These factors can compromise cellular proteostatic mechanisms such as autophagy and contribute to the development or progression of age-related neurodegenerative disorders, such as Alzheimer’s, Huntington’s or Parkinson’s disease, and amyotrophic lateral sclerosis [134,135,136,137,138].

Research at the interface of autophagy and ageing highlights interesting and elegant aspects of this interplay [139]. Lifespan extension can be achieved with tissue-specific overexpression of a single *atg* gene, revealing the minimum intervention with sufficient effect in organismal level. Furthermore, autophagy stimulation in a select tissue can have systemic effects and influence ageing in a cell-non-autonomous manner. Finally, targeting and clearance of specific dysfunctional components via selective types of autophagy may be sufficient for longevity, highlighting the importance of selectivity and avoidance of excessive off-target energy-consuming autophagic activity. Focusing on research in the nematode *C. elegans*, we summarize recent advances in our understanding of the role of general and selective neuronal autophagy modulation in neuroprotection as well lifespan and healthspan regulation.

## 2. Autophagy and Ageing in *Caenorhabditis elegans*

### 2.1. Caenorhabditis elegans as a Model Organism

*C. elegans*, a self-fertilizing hermaphrodite nematode species, was the first multicellular organism with a complete genome sequence [140]. 60–80% of *C. elegans* genes have human homologues [141]. The ease of genetic manipulation have led to the molecular identification of many key genes in developmental and cell biological processes [142]. A tremendous number of advantages such as small size, large brood populations, rapid life cycle, ease of cultivation, low maintenance expense, long-term cryopreservation, invariant cell number and development, transparency, and the ability to reduce gene activity using feeding RNAi render *C. elegans* an outstanding experimental system [143]. Primarily ethical concerns and secondarily human life expectancy and feasibility direct research to non-human model organisms [144]. Due to relatively short life expectancy, a wide range of biological processes can be easily assessed during the whole life span of the organism. In order to study the role and function of a specific protein of interest and further model the phenotype deriving from a human disease, researchers either mutagenize the respective homologue gene or overexpress the human gene implicating in the disease ubiquitously or in a specific tissue of *C. elegans* [145,146]. Expression of specific human proteins linked to neurodegeneration (e.g., α-synuclein, amyloid-β peptide or tau) leads to cellular toxicity in worms and flies. At least to some extent, invertebrate systems give the potential to generate important findings regarding mechanisms of neurotoxicity, which are extended in research in mammalian systems. Noteworthy, *C. elegans* nervous system structure and connectome is fully characterized, providing the most complete wiring reconstitution of any nervous system [147,148,149].

No perfect model organism exists to absolutely mimic human biology. Despite the high degree of conservation, there is still evolutionary distance between *C. elegans* and humans. Some mammalian genes have no homologs in the nematode (for example genes in the regulatory cascade of Hedgehog signaling) [150]. Furthermore, physiologically important systems are not found in the nematode, such as the immune system. The small size of *C. elegans* causes a certain difficulty in obtaining large amounts of nematodes at late ages in order to be used for the assessment of LGG-1 (LC3, GABARAP and GATE-16 family-1) lipidation for example. However, challenging methods for the cultivation of large numbers of aged worms have been developed and both protein and mRNA levels are estimated as a function of age [151,152,153].

### 2.2. Autophagy in Caenorhabditis elegans

The use of animal models is essential for understanding the role of autophagy in development, cell death, and multiple pathologies throughout an entire lifespan. Various nematode models of autophagy-related human pathologies, such as neurodegeneration, metabolic disorders, immune and infectious diseases exist [154]. In *C. elegans* autophagy occurs in many cell types and plays an essential role in physiology and development, such as survival under nutrient deprivation, removal of protein substrates, degeneration of 6 touch receptor neurons caused by toxic ion-channel variants, dauer formation, prevention of bacterial infection and ageing [155,156,157,158,159,160,161,162]. Genetic studies utilizing the aggrephagy pathway during embryogenesis revealed essential autophagy genes [162,163]. High conservation of autophagy genes and respective molecular mechanisms between *C. elegans*, yeast and mammals paved the way for the use of the nematode to study autophagy under the context of development, ageing, and pathophysiology (Table 1). Approaches to monitor and study autophagy in *C. elegans* are constantly expanding [164,165,166].

Emerging evidence implicate neuronal selective autophagy in the development of neurodegenerative diseases [199]. A relatively high degree of redundancy of SARs not only denote the importance of selective autophagy but also render the study of selective autophagy more complex and challenging. For example, p62/SQSTM1 (sequestosome 1), NBR1 (neighbor of BRCA1 gene 1 autophagy cargo receptor), TAX1BP1 (Tax1 binding protein 1), NDP52 (52-kDa nuclear dot protein), OPTN (optineurin), TOLLIP (toll interacting protein), ATG16L1, ALFY (autophagy-linked FYVE) and TRIM5 (tripartite motif containing 5) serve as selective aggrephagy receptors; BNIP3 (BCL2 interacting protein 3), NIX (Nip3-like protein X), FUNDC1 (FUN14 domain containing 1), BCL2L13 (BCL2 like 13), FKBP8 (FK506-binding protein 8), PHB2 (prohibitin 2), NIPSNAP1/2 (4-nitrophenylphosphatase domain and non-neuronal SNAP25-like proteins 1 and 2), NPD52, OPTN, AMBRA1 serve as selective mitophagy receptors and FAM134B (family with sequence similarity 134 member B), Sec62 (SEC62 homolog, preprotein translocation factor), RTN3 (reticulon 3), CCPG1 (cell cycle progression 1), ATL3 (atlastin GTPase 3) and TEX264 (testis expressed 264) serve as selective ER-phagy receptors (reviewed in [199]). Studies in lower organisms with less developed SAR (but also ATG8 family) repertoire such as *C. elegans* can serve as ideal abstract model in dissecting key features and roles of selective autophagy.

Inefficient or impaired autophagy has been linked to neurodegenerative disorders, such as Parkinson’s, Alzheimer’s and Huntington’s disease. However, it has been reported that autophagy effect on neuronal integrity is developmental-stage dependent. While increased levels or intact autophagy in ageing cells and organisms render enhanced health span and longevity, in early developmental life can be harmful [200]. Such a model of antagonistic pleiotropy effect of autophagy during development and ageing can be easily studied utilizing *C. elegans* under standard laboratory conditions.

### 2.3. Interplay between Autophagy and Ageing in Caenorhabditis elegans

In *C. elegans*, it is feasible to monitor and study age-dependent accumulation of immature autophagosomes and decreased autophagic degradation across all tissues in vivo over the organismal lifetime [201]. Long-lived mutants, such as *daf-2* [impaired insulin/IGF-1 (insulin-like growth factor-1) receptor signaling], *let-363/TOR(RNAi)* (impaired TOR signaling), *eat-2* (diet restriction), *clk-1* (mitochondrial respiration deficiency) and *glp-1* (germline-deficiency), require autophagy and exhibit increased puncta levels of the GFP (green fluorescent protein) tagged ortholog of human GABARAPL1, LGG-1, as well as increased expression of several autophagy genes [156,180,202,203,204]. Extended longevity of *daf-2* mutant worms is *bec-1*-, *atg-7-*, *atg-12-* and *lgg-1*-dependent [155]. *daf-2* mutants also require the transcription factor DAF-16/FOXO (abnormal dauer formation-16/forkhead box O) for enhanced longevity, which promotes the upregulation of *atg* genes in worms, flies and mammals [122,205,206]. During age-related autophagy dysregulation, inhibition of early steps of autophagosome nucleation via inactivation of *pha-4* (defective pharynx development-4) or *bec-1* leads to improved muscle integrity, neuroprotection, healthspan and lifespan [207].

## 3. Neuronal Autophagy in Ageing: Insights from *Caenorhabditis elegans*

### 3.1. Autophagy Induction

In eukaryotes, AMPK is the nutrient and energy sensor that functions to maintain cellular energy homeostasis [208,209]. AMPK regulates autophagy by at least two main modes of action. Firstly, via direct phosphorylation of mammalian TOR-binding partner raptor, mediating mTOR pathway inhibition [210]. Secondly, via direct phosphorylation of ULK1 [211,212]. Apart from the above roles in autophagy regulation, AMPK plays a role in ageing as well [213]. In *C. elegans*, increased gene dosage or activation of AAK-2 (AMP-activate kinase-2) catalytic α subunit, extends lifespan [214,215,216,217,218]. In the case of diet restriction-induced longevity, a single pair of sensory neurons in the head (ASIs) act cell non-autonomously to signal in peripheral non-neuronal body tissues to increase metabolic activity [219].

Ectopic expression of ANMT-1/NNMT (amine *N*-methyltransferase-1/nicotinamide *N*-methyltransferase) maintains neuronal function in old wild type and various disease model worms, affecting longevity as well [220]. When NNMT is ectopically expressed in human and rat neuronal cells exerts neuroprotective effects through ephrin B2 and Akt signaling, regulating energy homeostasis. ANMT-1 competes with LCMT-1 (leucine methyltransferase homolog-1) for methyl groups from SAM (S-adenosyl-methionine) [221,222]. Under physiological cellular SAM levels, LCMT-1 activity leads to autophagy inhibition through LET-92/PP2CA (lethal-92/protein phosphatase 2 catalytic subunit alpha) and NPRL-2/NPRL2 (nitrogen permease regulator like homolog-2/NPR2-like, GATOR1 complex subunit). When ectopically expressed, ANMT-1 methylates NAM (nicotinamide) to MNA (*N*-methylnicotinamide). The reaction is irreversible causing SAM and therefore methyl groups scarcity [223]. LCMT-1 is unable to elicit its physiological inhibitory activity. Therefore, autophagy is activated and neuronal function is ameliorated in ageing worms. On the other hand, in younger worms, ANMT-1 activity caused abnormal behavior, disturbing neuronal homeostasis and dopamine signaling, possibly due to excessive autophagy. The above example shows that ectopic genetic interventions obey to the universal antagonistic pleiotropic theory as well. Age-correlated studies in model organisms along with biochemical, genetic and cellular observations allows in depth understanding of naturally fine-tuned biological processes in organismal lifespan dimension.

### 3.2. PI3K Complex and PI3P

Autophagic activity decreases with animal ageing in many species including *C. elegans* [201]. Age-related decline of autophagy takes place in several tissues such as intestine, body-wall muscle, pharynx, and neurons of wild type animals. Autophagy is considered as a convergent process of several longevity interventions including calorie restriction, insulin/IGF-1 or TOR signaling inhibition, germline removal and reduced mitochondrial respiration [155,156,158,160,203]. Rubicon (run domain Beclin-1 interacting and cysteine-rich containing protein), an autophagy related protein that binds to the PI3K complex and negatively regulates autophagy, inhibits both autophagosome-lysosome fusion and endocytic trafficking [224,225]. Age-dependent increase of Rubicon expression in worms, flies and mice, impairs autophagy over time, causing animal healthspan decline [226]. Knockdown of Rubicon specifically in neurons has the greatest effect on lifespan extension comparing to hypodermal or intestinal knockdown. Furthermore, Rubicon knockdown ameliorates several age-associated phenotypes, such as polyglutamine aggregation in body wall muscles, decline in locomotion and oxidative stress resistance in an autophagy-dependent manner, rendering increased expression of Rubicon an autophagy-related signature of ageing.

DAF-2/IGF-1 (abnormal dauer formation-2/insulin-like growth factor-1) and its major transcription factor target DAF-16/FOXO regulate longevity in *C. elegans* [227,228,229]. ATG-18/WIPI2 sufficiently mediates the DAF-2 longevity signal. It acts specifically in chemosensory neurons and intestinal cells of *C. elegans* to maintain lifespan and respond to dietary restriction, in a cell non-autonomous manner, through the release of neurotransmitters and neuropeptides [181].

### 3.3. ATG8

During *C. elegans* development LGG-1 and LGG-2, share similar expression and localization pattern. However, GFP::LGG-2 is more abundant in some head and tail neurons and the neuron cell bodies of the ventral nerve cord where might play a particular role [185]. As reported for LC3 in mammalian cells, autophagic flux can be measured in whole animals or specifically in a particular tissue of *C. elegans* utilizing LGG-1 tandemly tagged with two fluorescent proteins at the N’ terminus (Cerulean-Venus) [230,231]. Release of a monomeric fluorescent protein upon arrival at the lysosome shows increased autophagic flux primarily in intestine, hypodermis and muscle cells and to a smaller extend in pharynx and neurons on day 5 animals compared to first-stage larvae.

Monitoring autophagic vesicle formation from day 1 to day 10 of adulthood of *C. elegans* using transgenic animals expressing either *gfp::lgg-1* or *mcherry::gfp:lgg-1* reporters indicate that the steady-state number of autophagosomes increases with age in intestine, muscle, pharynx and neurons, albeit with different trajectories [201]. Interestingly, the steady-state number of autolysosomes increases until day 3 to 5 but then somehow stagnate in intestine, muscle and pharynx whereas the number of autolysosomes decreases over time in neurons. The increased number of autophagosomes, which is not accompanied by increased number of autolysosomes, might reflect an age-dependent decline in autophagic activity in those major tissues. The above decline occurs at a step after autophagosome formation. In the same study, it is suggested that neuronal autophagic activity of the long-lived *daf-2* insulin/IGF-1 receptor mutant or the germline-less *glp-1* Notch receptor mutant is higher in early adulthood compared to wild type, whereas age-related impairment of neuronal autophagic activity takes place over mid-life similarly to wild type worms.

Functional neuronal autophagy is critical for the maintenance of normal axonal homeostasis and prevention of axonal degeneration under physiological and stress conditions [232,233,234,235]. Axonal damage responses include calcium influx, membrane sealing, activation of injury signaling, changes in transcription and autophagy activation [236,237,238,239]. Specifically, autophagy stabilizes microtubules, promotes axon regeneration and improves locomotor functional recovery in mice after spinal cord injury [240]. In addition, axon injury upregulates the autophagy genes *Ambra*, *Atg5*, *Beclin 1* and *LC3* in the central and peripheral nervous system [240,241]. In *C. elegans*, single neuron axotomy is feasible using a variety of lasers [242,243,244,245]. Tandem fluorescent-tagged LGG-1 monitoring during axon regeneration reveals increased number of autophagic vesicles [246]. The capacity of animals for axon regeneration and autophagy activation after axonal injury undergo an age-dependent decline. Autophagy-activating agents fail to enhance axon regrowth in young PLM neurons but interestingly succeed to partially rescue these defects in old neurons. Furthermore, autophagy activation after injury depends on DLK-1/MAP3K12 (dual-leucine zipper kinase-1/mitogen-activated protein kinase kinase kinase 12) and LIN-12/NOTCH4 (Ca^2+^ signaling. Abnormal cell lineage-12/notch 4 receptor), identified previously as an inhibitor of axon regeneration, colocalizes with autophagic vesicles in PLM neurons suggesting that LIN-12 might be a target of autophagy during axon regeneration [246,247,248]. This example suggests that pharmacological activation of autophagy in aged animals can specifically enhance neuronal axon regeneration boosting the age-attenuated regrowth capacity.

Young adult animals expressing amyloid-β_3-42_ or polyglutamine (Q_40_) in neuronal cells show increased levels of LGG-1 and moderate increased levels of SQST-1 (sequestosome 1)/p62 via immunodetection, indicating impairment of autophagy [249]. Whether such an increase represents transcriptional induction of LGG-1 and SQST-1 was not examined.

β-amyrin, a pentacyclic triterpene found in many medicinal plants, shows a protective effect on dopaminergic neurons by reducing dendrite blebbing or rounding induced by 6-OHDA (6-hydroxydopamine) [250]. In addition, β-amyrin reduces α-synuclein aggregation in muscle cells, upregulates *lgg-1* mRNA levels and increases the number of GFP::LGG-1 puncta in seam cells.

### 3.4. SQST-1/p62

In *C. elegans*, SQST-1 is required for autophagosome formation in neurons, rather than for increasing the rate of (or facilitating the) autophagosomal turnover [251]. *sqst-1* mutant animals display a tissue-specific block in autophagy in nerve-ring neurons. Unlike core autophagy genes, *sqst-1* is not implicated in other conserved longevity paradigms, including *daf-2*/insulin/IGF-1 receptor and germline-less *glp-1* mutants or animals with reduced levels of mTOR and reduced levels of components of the mitochondrial electron transport chain (*let-363* and *cyc-1* RNAi, respectively) [139]. However, *sqst-1* is required under specific, potentially proteotoxic conditions, such as a hormetic heat shock. In contrast to wild type animals in which a hormetic heat shock reduces aggregation, *sqst-1* mutant animals expressing neuronal polyglutamine show an increased aggregate load in neurons following a hormetic heat shock [251,252]. Both hormetic heat shock- and HSF-1 (heat shock factor-1)-mediated survival require the autophagy genes *unc-51*, *bec-1* and *lgg-1* as well. SQST-1 overexpression is sufficient to induce neuronal (and intestinal) autophagy and results in lifespan extension in an autophagy-dependent manner. Interaction of SQST-1 with ubiquitinated proteins is required for SQST-1 overexpression lifespan extension. Overexpression of SQST-1 results in lifespan extension and improved proteostasis in *C. elegans* strains with protein-folding defects or under proteotoxic conditions as well. Specifically, while the number of neuronal polyglutamine aggregates is unchanged, the mobility of both the aggregated and the diffuse polyglutamine proteins is increased upon overexpression of SQST-1.

### 3.5. Autophagosome Formation

A systematic analyses utilizing genetic ablation of ATG proteins and markers for synaptic vesicle clustering, cytoskeleton organization and active zone position in *C. elegans*, revealed the role of 18 distinct autophagy genes in neuronal development [253]. Specifically, ATG proteins covering all the stages from autophagosome biogenesis until maturation are required for clustering of the synaptic vesicle proteins RAB-3 and SNB-1 (synaptobrevin-1) in AIY interneurons during development. In contrast, the selective autophagy genes *atg-11*, the nematode specific *epg-2* and *sqst-1* do not show AIY presynaptic defects. Retention of the respective rescuing array for either *atg-9*, *lgg-1*, or *atg-2* mutants in AIY neurons results in rescue of the AIY presynaptic defects. Retention of the same rescuing array in other neurons (including postsynaptic partner RIA), but not in AIY neurons, does not result in rescue of the AIY presynaptic defects. Therefore, autophagy acts in a cell-autonomous manner in AIY neurons to promote synaptic vesicle clustering. Additionally, defects in autophagy affect different elements of presynaptic assembly, namely the localization of F-actin and the active zone protein SYD-1 (synapse defective protein-1) to presynaptic sites. However, autophagy is not required for the development of the glia that specifies the synaptic positions in AIY. Autophagosomes are present in cell bodies and near presynaptic sites of developing AIY and nociceptive sensory PVD neurons in *C. elegans* embryos when axon outgrowth and synaptogenesis occur. Endogenous ATG-9 in both adults and 3-fold embryos is present pan-neuronally. Moreover, ATG-9 is enriched in presynaptic regions and colocalizes with RAB-3 in AIY neurons in an UNC-104/KIF1A (uncoordinated-104/kinesin family member 1A)-dependent manner. Similarly, punctate ATG-9 colocalizes with RAB-3 in nociceptive sensory PVD neurons. Autophagy deficiency in distinct steps of the process leads to significantly longer outgrowth of the nociceptive sensory PVD axon in L3, L4 and adult worms.

Presynaptic neurons can regulate synaptic clustering of postsynaptic muscle cell receptors selectively through autophagy [108]. Loss of GABA- and acetylcholine-dependent innervation causes increased autophagy in muscle cells and autophagosome engagement of GABA_A_ receptors after endocytosis from cell membrane. On the contrary, under the same condition, acetylcholine receptors do not show any autophagosome localization in the same cells, indicating that autophagy targets GABA_A_ receptors selectively. The above mechanism provides control of GABA_A_ inhibitory synapse activity while leaves acetylcholine excitatory synapse activity unaffected through autophagy.

The atypical E3 ubiquitin ligase, RPM-1/MYCBP2 (regulator of presynaptic morphology-1/MYC binding protein 2) restricts UNC-51 activity and furthermore autophagosome formation specifically at the axon termination sites affecting initiation of autophagy in mechanosensory neurons [254]. Worms lacking *rpm-1* show elevated UNC-51 levels in axon termination sites, which causes excessive autophagy initiation and autophagosome formation. The above leads to abnormal axon termination, synapse maintenance and behavioral habituation.

Among several toxic protein aggregates, autophagy plays a critical role in removal of tau [255,256,257,258]. Axon injury-induced autophagy and furthermore axon regeneration capacity are reduced in transgenic *C. elegans* expressing the highly amyloidogenic tau species F3ΔK280, but not the anti-aggregant tau (with I277P and I308P introduction) [259,260]. In addition, pro-aggregant tau impairs autophagic vesicle trafficking across neuron axons. Autophagy inducers reduce the accumulation of misfolded and aggregated proteins, attenuating the spreading of tau and neuronal damage [259,261,262,263,264,265,266,267]. Either rapamycin treatment or axon-injury is sufficient to enhance the number and trafficking of autophagic vesicles in young wild type PLM neurons. In contrast, neither rapamycin nor axon injury after axotomy was able to induce the formation or trafficking of autophagic vesicles in young PLM neurons of the tauopathy model [260]. However, it would be of particular interest to compare autophagy responses of the above experimental set during ageing. Interestingly, loss of the *C. elegans* homolog of tau, PTL-1 (protein with tau-like repeats-1), leads to increased basal level of autolysosomes and impaired autophagy activation after injury. Therefore, tau depletion as well as aggregation modulate autophagy responses in neurons after injury. It is not clear whether autophagy-lysosome system impairment is a contributor or a consequence of tauopathies [268,269].

Rho family small GTPases and furthermore actin cytoskeleton regulate neuronal axonogenesis and morphogenesis [270]. In *C. elegans* the small GTPase CED-10/RAC1 (cell death abnormality-10/Rac family small GTPase 1) has been associated with impaired autophagy and dopaminergic neuron death through α-synuclein accumulation [271].

Infections by pathogenic microbes are suspected to trigger functional neuronal ageing through increased expression of immune molecules [272,273,274]. Some AMPs (antimicrobial peptides) were suggested to have non-antimicrobial functions in the neurons [275,276]. Age-associated expression of NLP-29 (neuropeptide-like protein-29), an AMP expressed in hypodermis, causes dendrite degeneration via the orphan GPCR (G protein-coupled receptor) NPR-12 (neuropeptide receptor-12) and autophagy induction in PVD neurons of *C. elegans* [277]. The same NLP-29/NPR-12 pathway causes dendrite degeneration through autophagy in rat cortical neurons suggesting evolutionally conservation.

Polyamine supplementation or inhibition of polyamine catabolism (which leads to increased intracellular polyamine levels) extends lifespan and rescues worms from age-associated neurodegeneration derived from manganese toxicity in an autophagy-dependent manner [278,279]. The above neuroprotective effect of polyamines is proposed to be exerted through translational regulation.

### 3.6. TFEB

TFEB (transcription factor EB) controls the expression of genes involved both in the lysosomal biogenesis and function and in major steps of autophagy, including autophagosome formation and autophagosome-lysosome fusion [280]. TFEB dysregulation is associated with neurodegenerative diseases and TFEB overexpression promotes neuroprotection [281,282,283,284]. Heterozygous loss-of-function mutations of the neurodegenerative disease protein PGRN (progranulin) result in TDP-43 (TAR DNA binding protein-43) inclusions and cause frontotemporal lobar degeneration [285,286,287]. Loss of both gene alleles leads to the lysosomal storage disease, neuronal ceroid lipofuscinosis [288]. In *C. elegans*, granulins, the cleavage products of progranulin, accumulate in the endolysosomal compartment and impair lysosomal function during ageing [289]. Furthermore, granulin expression may result in neuronal dysfunction and granulin-expressing animals show impaired short-term associative learning. Disrupted protein homeostasis causes nuclear translocation of HLH-30 (helix-loop-helix-30)/TFEB, in order to induce expression of genes involved in lysosomal function and autophagy, as well as progranulin.

### 3.7. Mitophagy

Mitophagy, the selective degradation of damaged or superfluous mitochondria by autophagy, is implicated in neurodegeneration and ageing [290,291,292]. Age-related mitochondrial dysfunction or damage play a prominent role in Alzheimer’s disease [293,294,295]. Expectedly, mitophagy is impaired in Alzheimer’s disease *C. elegans* models [296]. In both amyloid-β and tau model nematodes, mitophagy enhancement reverses memory impairment through PINK-1 (PTEN-induced kinase-1)-, PDR-1 (Parkinson’s disease related 1)/-, or DCT-1 (DAF-16/FOXO controlled, germline tumor affecting-1)-dependent pathways. Patients carrying different FTD (frontotemporal dementia) tau mutations show diverse pathological features [297,298,299]. The toxicity of different tau mutations on neuronal mitophagy in *C. elegans* varies as well. The FTD P301L mutant tau is more detrimental than wild type tau. Animals expressing the V337M mutant tau (another mutation found in familial cases of FTD) display an intermediate mitophagy impairment in neurons in response to NaN_3_ [300].

Overexpression of UCP-4/SLC25A27 (uncoupling protein-4/solute carrier family 25 member 27) ameliorates neuronal integrity during ageing in *C. elegans* [301]. Animals overexpressing UCP-4 in mechanosensory touch receptor neurons show decreased mitochondrial membrane potential as well. Mitophagy-deficient animals overexpressing UCP-4 fail to attenuate age-dependent neuronal defects. The above results, derived from a protein overexpression, resemble the effects reported for the activity of mitochondrial uncoupling agents and mitophagy inducers such as CCCP (carbonyl cyanide m-chlorophenyl hydrazone) and DNP (2,4-dinitrophenol).

### 3.8. Aggrephagy

Autophagy genes are employed in the degradation of protein aggregates. Distinct autophagy mutants exhibit different patterns of accumulation of SQST-1 aggregates in different tissues and developmental stages of *C. elegans* [302]. *ATG* genes and the metazoan-specific *epg-3*, *epg-4* and *epg-5* genes, are required for degradation of SQST-1 in all tissues in embryos. Loss of function of core autophagy genes causes differences in SQST-1 accumulation at the larval and adult stages. The *lgg-1* mutant shows the strongest defect in SQST-1 clearance. *epg-3* and *epg-4* mutants cause weaker defect in SQST-1 degradation at larval and adult stages. *epg-3* is essential for SQST-1 removal in the hypodermal cells, while mild defect is monitored in the body wall muscle cells, neurons and intestine. *epg-4* mutants exhibit weak defects in all tissues. *epg-5* mutants show a weak autophagy defect in hypodermal cells at the embryonic stage. Finally, loss of function of *calpain-2* (*clp-2*) causes accumulation of SQST-1 aggregates in hypodermis and neurons at larval and adult stages. In contrast, degradation of SQST-1 in body wall muscle and intestine is normal.

Posttranslational modification of the human mutant Huntingtin by acetylation at lysine residue 444 shows improved clearance of the mutant protein in chemosensory neurons of *C. elegans* and may prevent cellular dysfunction and neurodegeneration in Huntington’s disease [303]. While mutant Huntingtin expression results in degeneration of chemosensory neurons, co-expression of CBP (CREB-binding protein) results in neuroprotection. Such a neuroprotection activity is abolished when CBP is co-expressed along with the acetylation-resistant mutant Huntingtin, suggesting that CBP-mediated neuroprotection in *C. elegans* requires intact lysine 444.

## 4. Concluding Remarks

Studies in long-lived models of *C. elegans* provide evidence for the important role of multiple *ATG* genes, establishing a link between autophagy and ageing (Table 2). It is becoming obvious that differential regulation of autophagy utilizes multiple ways to increase lifespan in a cell-autonomous or cell-non-autonomous manner. Autophagy declines in a tissue- and type-specific manner upon normal ageing. One possible explanation for the above observation could involve tissue-specific increased activity of the nutrient sensor mTOR upon ageing, which negatively regulates autophagy [304,305]. In addition, ageing negatively affects several steps of the autophagic process and lysosome activity. These include autophagosome transport in neurons, autophagosome accumulation in *C. elegans* and mice livers, lysosomal acidification in yeast and lysosomal protease activity in *C. elegans* [117,201,207,306,307,308]. Neuronal cells have developed unique and unconventional mechanisms to regulate autophagy. Spatiotemporal regulation of autophagy is implicated in neurotransmitter release, neuronal plasticity and behavioral outputs. A plethora of research avenues is open for future investigation. The emerging interest in the selectivity of autophagic cargo in neurons dictates the need for development of novel reporters for monitoring selective autophagy in vivo and in vitro. Since, it is known that neuronal activity and plasticity modulate the proteasomal system, correlative monitoring of autophagy and proteasome activity would gain insight in neuronal proteostasis regulation upon ageing [309,310,311]. Non-canonical roles of autophagy with possible link to ageing deserve further elucidation, such as the molecular and functional distinction between secretory and degradative autophagosomes in neurons. Organelle isolation and purification such as autophagosomes from different neuronal populations and compartments would shed light into age-dependent alterations in canonical and non-canonical functions of autophagy. Finally, autophagy may serve as a molecular and vesicular platform for pharmacological and genetic interventions to target specific substrates in a cellular/tissue- and temporal-specific manner.

## Figures and Tables

**Table 1:** Evolutionarily conserved autophagy-related genes of *Caenorhabditis elegans*.

	*C. elegans*	Mutant Allele	*Homo Sapiens*	Reference
Initiation/ ULK1 complex	*unc-51*	*e369*	*ULK1, ULK2*	[108,155,157,167]
	*epg-1*	*bp417*	*ATG13*	[168]
	*epg-7*	*tm2508*	*RB1CC1 (FIP200)*	[169]
	*epg-9*	*bp320*	*ATG101*	[170]
Nucleation/ PI3KC3 complex	*epg-8*	*bp251, ok2561*	*ATG14*	[171]
	*bec-1*	*ok691, ok700, bp613*	*BECN1*	[155,172,173,174,175,176]
	*vps-15*	*or1235, ok3132*	*PIK3R4 (VPS15)*	[177]
	*vps-34*	*h741*	*PIK3C3*	[178]
Phagophore formation/ PI3P binding	*atg-2*	*bp576*	*ATG2A, ATG2B*	[179]
	*atg-9*	*bp564*	*ATG9A, ATG9B*	[179]
	*atg-18*	*gk378, bp594*	*WIPI1, WIPI2*	[162,176,180,181]
Phagophore formation/ ATG12 conjugation system	*atg-5*	*bp484*	*ATG5*	[182]
	*atg-7* *	*bp422, bp290*	*ATG7*	[155,174,176,179,183]
	*atg-10*	*bp421*	*ATG10*	[183]
	*lgg-3*	*tm1462*	*ATG12*	[183]
	*atg-16.1*	*gk668615*	*ATG16L1*	[182]
	*atg-16.2*	*bp636, ok3224*	*ATG16L2*	[176,182]
	*epg-6* **	*bp242*	*WDR45B (WIPI3), WDR45 (WIPI4)*	[179]
Phagophore elongation/ LC3 conjugation system	*atg-3*	*bp412*	*ATG3*	[183]
	*atg-4.1*	*bp501*	*ATG4A, ATG4B*	[184]
	*atg-4.2*	*tm3948*	*ATG4C, ATG4D*	[184]
	*lgg-1*	*bp500, tm3489*	*GABARAP, GABARAPL1, GABARAPL2*	[155,162,185,186]
	*lgg-2*	*tm5755*	*MAP1LC3B*	[185,186,187]
Autophagosome- lysosome fusion	*lmp-1*	*nr2045*	*LAMP1*	[188,189]
	*snap-29*	*tm2060*	*SNAP29*	[180,190]
	*syx-17*	-	*STX17*	[191]
	*vps-39*	*tm2253, ok2442*	*VPS39*	[186]
Negative regulation of autophagosome assembly	*epg-2*	*bp287, bp444*	-	[168]
	*epg-3*	*bp405*	*VMP1*	[162,168]
	*epg-4*	*bp425*	*EI24*	[162,168]
	*epg-5*	*bp450, tm3425*	*EPG5*	[162]
Autophagy gene regulation	*let-363*	*h98, h111*	*MTOR*	[158,160,192,193]
	*vps-41*	*ep402*	*VPS41*	[186]
Autophagy receptors	*sepa-1*	*bp456*	-	[168]
	*sqst-1*	*ok2892*	*SQSTM1 (p62)*	[169]
Endosome transport	*rab-7*	*ok511, tm3300*	*RAB7A*	[185,186,194,195,196]
	*rab-10*	*ok1494*	*RAB10*	[197,198]

Human gene and previous/alias (in parentheses) approved symbols according to HUGO Gene Nomenclature Committee (https://www.genenames.org/ accessed on 20 March 2021). * Participates at LC3 conjugation system as well. ** Participates at phagophore elongation as well.

**Table 2:** Regulation of ageing by neuronal autophagy in *Caenorhabditis elegans*.

Effector	Intervention	Mechanism	Effect	Reference
AAK-2	Increased gene dosage or activation	Autophagy initiation	Lifespan extension	[214,215,216,218,219]
ANMT-1	Neuronal expression	Autophagy initiation	Lifespan regulation in aged worms	[220]
Rubicon	Neuronal knockdown	Autophagosome-lysosome fusion/ endocytic trafficking	Lifespan extension	[226]
ATG-18	Mutant	Neuronal and intestinal autophagosome formation	Dietary restriction- and IGF-mediated longevity	[181]
DLK-1, LIN-12	Axon injury	Neuronal autophagic degradation	Enhanced neuronal axon regeneration upon autophagy induction in aged worms	[246,247,248]
β-amyrin	Administration	Autophagy induction	Neuroprotection	[250]
SQST-1	Neuronal overexpression	Autophagosome formation	Lifespan extension	[251]
ATG-9, LGG-1, ATG-2	Rescue in AIY neurons	Synaptic vesicle clustering	Rescue of AIY presynaptic defects	[253]
-	Muscle innervation loss	Autophagosome engagement of GABA_A_Rs	Postsynaptic clustering regulation	[108]
RPM-1	Mutant	Excessive autophagosome formation	Abnormal axon termination	[254]
-	Axon injury	Impaired autophagy in tauopathy model	Impaired axon regeneration	[260]
CED-10	Mutant	Impaired autophagy	α-synuclein neuronal inclusions	[271]
NLP-29	Age-associated expression	Neuronal autophagy induction	Dendrite degeneration	[277]
Spermine	Administration	Transcriptional regulation of autophagy	Neuroprotection	[279]
Granulin	Endogenous expression	HLH-30 nuclear translocation	Impaired short-term associative learning	[289]
NAD^+^, urolithin A, actinonin	Administration	Mitophagy enhancement	Inhibited amyloid-β- and tau-mediated cognitive deficits	[296]
Tau	Pan-neuronal expression	Inhibition of mitophagy	Inhibited degradation of damaged mitochondria	[300]
UCP-4	Neuronal overexpression	Mitophagy stimulation	Attenuated age-dependent neurodegeneration	[301]
ATGs, EPGs, CLP-2	Mutants	Inhibition of aggrephagy	Tissue- and stage-specific clearance of SQST-1 aggregates	[302]
CBP	Neuronal overexpression	Autophagic clearance of huntingtin *	Neuroprotection	[303]

* Non-direct measurement.

## Data Availability

No new data were created or analyzed in this study. Data sharing is not applicable to this article.
